# Biology, role and therapeutic potential of circulating histones in acute inflammatory disorders

**DOI:** 10.1111/jcmm.13797

**Published:** 2018-08-07

**Authors:** Peter Szatmary, Wei Huang, David Criddle, Alexei Tepikin, Robert Sutton

**Affiliations:** ^1^ Liverpool Pancreatitis Research Group Royal Liverpool University Hospital and Institute of Translational Medicine University of Liverpool Liverpool UK; ^2^ Department of Cellular and Molecular Physiology University of Liverpool Liverpool UK; ^3^ Department of Integrated Traditional Chinese and Western Medicine Sichuan Provincial Pancreatitis Center West China Hospital of Sichuan University Chengdu China

**Keywords:** extracellular histones, immunothrombosis, inflammation, innate immunity

## Abstract

Histones are positively charged nuclear proteins that facilitate packaging of DNA into nucleosomes common to all eukaryotic cells. Upon cell injury or cell signalling processes, histones are released passively through cell necrosis or actively from immune cells as part of extracellular traps. Extracellular histones function as microbicidal proteins and are pro‐thrombotic, limiting spread of infection or isolating areas of injury to allow for immune cell infiltration, clearance of infection and initiation of tissue regeneration and repair. Histone toxicity, however, is not specific to microbes and contributes to tissue and end‐organ injury, which in cases of systemic inflammation may lead to organ failure and death. This review details the processes of histones release in acute inflammation, the mechanisms of histone‐related tissue toxicity and current and future strategies for therapy targeting histones in acute inflammatory diseases.

## 1 INTRODUCTION

Histones were first described by Albrecht Kossel in 1884 as histidine‐rich peptones derived from the nuclear component of avian red blood cells^1^; he was awarded the Nobel Prize in Physiology or Medicine for this and other work on the nucleus of cells in 1910. Histones are highly conserved across all eukaryotic cells,^2^ and act as nuclear chaperone proteins, interacting with nucleic acids due to their highly positive charge^3^ from lysine and arginine residues. Each nucleosome particle consists of 147 base pairs of DNA, wrapped in 1.7 turns around a protein octamer of core histones (H2A, H2B, H3 and H4), further compacted by linker histones (H1 and/or H5).^4^

Numerous post‐translational modifications of histones have been identified, including acetylation, methylation, phosphorylation, ubiquitylation, sumoylation, ADP ribosylation, deimination and proline isomerization.^5^ In normal cell function, these alter the nature of the histone‐DNA interaction and allow transcription to occur. More recently, controlled histone degradation has been described in neutrophils leading to chromatin decondensation and release of genomic DNA laced with granular proteins as neutrophil extracellular traps (NETs).^6^, ^7^ These meshwork‐like structures promote intravascular thrombosis,^8^ limit spread of microorganisms, encourage cancer metastasis^9^ and cause direct injury to adjacent cells.^10^

This review details what is known about the role of histones as alarmins or DAMPs (damage‐associated molecular patterns), processes leading up to active histone release as principle components of NETs, mechanisms of injury related to extracellular histones and therapeutic strategies for histone detoxification in acute inflammatory conditions.

## 2 HISTONES AS DAMPS

Among the earliest recognized and better‐described ways in which histones exacerbate cellular injury is in their role as alarmins or DAMPs. Histones released passively from necrotic cells (or actively by other modes of cell death including NETosis) act on adjacent cells and circulating immune cells via pattern recognition receptors to effect specific biological activity. In in vivo systems, these effects can be difficult to study, as histones are co‐released with nuclear DNA and other nuclear DAMPs such as HMGB1 (high mobility group box protein 1), each with their individual activities. Indeed, the mechanism of cell necrosis has significant impact on the kinetics of nuclear DAMP release,^11^ and nuclear DAMPs acting as complexes have been reported to exert different activities compared to protein isolates.^12^ Furthermore, where purified histones injected into experimental animals are lethal within minutes,^13^ necrotic cell death releases nucleosomes (ie: histone‐DNA complexes) which overall appear to be less toxic.^14^ Indeed a study injecting similar doses of nucleosomes in mice makes no mention of toxicity,^15^ and others have demonstrated cofactors such as HMGB1 responsible for the immune‐stimulatory effects of nucleosomes.^16^ Only through the interplay of plasma proteases and nucleases including DNAse1 and factor VII activating protease does nucleosome decondensation occur^17^; however, this also degrades the histone component and limits cytotoxicity.^18^ These effects may have significant implications for in vitro signalling studies using recombinant proteins, as effects of isolated nucleosome components may not become apparent in this setup.

Fragments of cell membrane and nuclear proteins also interact with complement proteins and complement cascade regulators to facilitate cell turnover and clearance.^19^ An important regulator of nucleosome toxicity appears to be factor H of the family of complement regulator proteins. Factor H is actively internalized by apoptotic cells, where it leads to C3 complement activation and cell surface expression, as well as enhanced nucleosome clearance and phagocyte cytokine‐release response to nucleosomes.^20^ Cells undergoing secondary necrosis can thereby elicit a targeted pro‐inflammatory response.^21^

**Figure 1:**
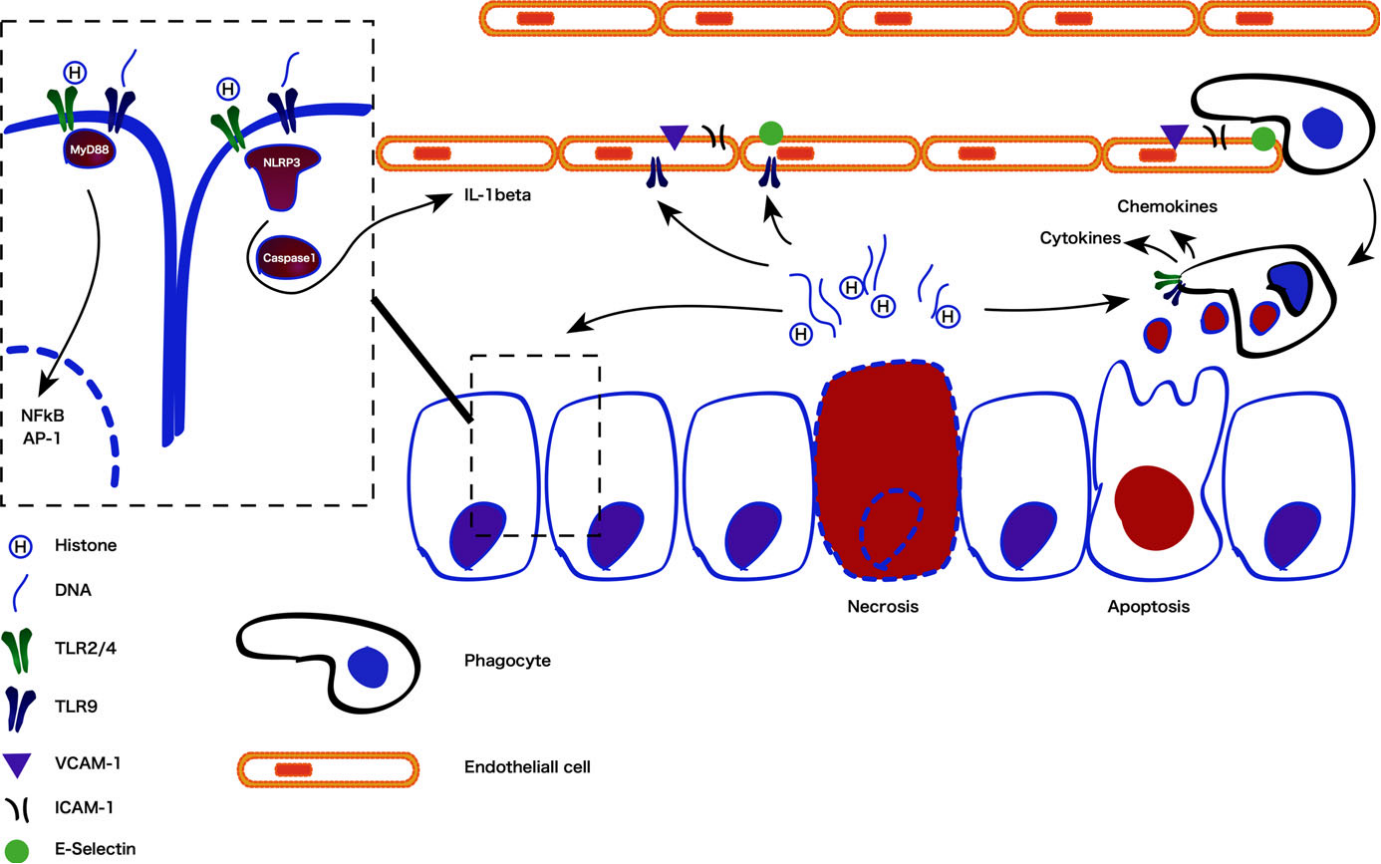
Immunostimulatory effects of passively released histones acting as damage‐associated molecular patterns

Once released from the nucleosome, extracellular histones exert their injurious effects in three ways summarized in Figure 1: (a) by acting as chemokines or inducing chemokine release; (b) by inducing cytokine release and/or apoptosis of adjacent cells and leukocytes; and (c) through direct cytotoxicity.

### 2.1 Chemoattractant effects

Histones are both directly chemoattractant, induce release of chemokines from adjacent cells and induce activation of vascular endothelium to promote adhesion and trans‐migration of leukocytes. Direct chemoattractant effects have been demonstrated in vitro using hepatocellular carcinoma cells. Histone‐induced migration of these cells is dependent on the activation of the MAPK/ERK/NF‐kB pathway via TLR4 (toll‐like receptor 4).^22^ Similarly, histones induce secretion of chemokines CXCL9 and CXCL10 from human monocytes and CXCL10 co‐localizes with extracellular histone H4 in necrotic (but not healthy) tissue.^23^ Extracellular histones H3 and H4 (but not H1 or H2A/H2B) also activate vascular endothelial cells to increase cell surface expression of E‐selectin, ICAM‐1 and VCAM‐1, thereby increasing leukocyte adhesion, rolling and transmigration in a TLR9‐dependent manner.^24^ In higher concentrations, these histones are toxic to the endothelium and represent a putative mechanism for pulmonary haemorrhage and ARDS in sepsis^13^ or pancreatitis.^25^ Histones acting on endothelial cells via TLRs 2 and 4 also activate NF‐kB and AP‐1 pathways to induce tissue factor expression,^26^ thereby creating a pro‐thrombotic milieu contributing to the microvascular thrombosis seen in many acute inflammatory diseases. Together, these effects describe the positive feedback loop that can lead to necroinflammation—where the death of relatively few cells induces further injury through inflammatory cell recruitment leading to organ failure, especially within the liver and/or kidney.^27^

### 2.2 Pattern recognition receptor responses

The intracellular signalling pathways of extracellular histones as DAMPs acting via TLRs2/4/9, MyD88, NF‐kB and the NLRP3 inflammasome have been well documented and recently reviewed.^28^, ^29^, ^30^ Functionally, histones injected into the renal artery of rats induced necroinflammation as well as IL‐6, TNF‐α and iNOS release.^31^ These effects were reduced in TLR2/4 knock‐out mice and more pronounced following LPS priming, which increased TLR2/4 mRNA transcription. Low doses of histone H3 (10 μg/mL) have been shown to induce release of IL‐6 and IL‐8 in ARPE‐19 cells, as well as lead to the phosphorylation of ERKs, p38 MAPK and JNK and inhibition of these kinases all resulted in reduced cytokine release.^32^ Higher doses (50 μg/mL), however, led to cell death in a manner that could not be inhibited using signalling kinase inhibitors. Histones also exacerbate ischaemia/reperfusion injury by a TLR9/MyD88‐dependent mechanism and enhance extracellular DNA‐mediated activation of TLR9 in immune cells.^33^ Further to their effect on TLRs, histones also appear to induce IL‐1β secretion and activation via an NLRP3/ASC/caspase1‐dependent mechanism, leading to neutrophil recruitment to sites of inflammation.^34^ Critically, induction of leukocyte cytokine production and release is not dependent on free, circulating histones; nuclear material within blebs from apoptotic cells can induce similar stimulatory effects within resident or infiltrating phagocytes.^35^

## 3 HISTONE PROCESSING AND ACTIVE RELEASE DURING NETOSIS

### 3.1 Signal recognition

A large number of different signals have been shown to be able to induce NET formation, including bacteria,^36^, ^37^ viruses,^38^ yeasts,^39^, ^40^ parasites,^41^ organic crystals,^42^ non‐organic matter,^43^ cytokines^44^ and cellular breakdown products including nuclear DAMPs.^45^, ^46^ In order to detect such a variety of signals, there is overlap and convergence of receptor pathways. This may explain some variability in early genetic knock out studies when defining which receptor is critical in mediating NET release. It would seem molecular pattern‐related NET release is mediated predominantly through TLRs 2, 4 and 9,^31^, ^46^, ^47^ immune complex‐related NET release is mediated via Fc receptors and MAC‐a^48^ and larger pathogens or inorganic matter lead to NETosis though size. The inability to phagocytose large particles within a given time appears to drive neutrophils to autodigest and release NETs in a process dependent on dectin‐1.^49^ While many signals leading to NETosis may make this an unlikely therapeutic target, it suggests that blocking destructive NETosis in sterile inflammation is possible without affecting a potentially beneficial antimicrobial response.

### 3.2 Signal transduction

**Figure 2:**
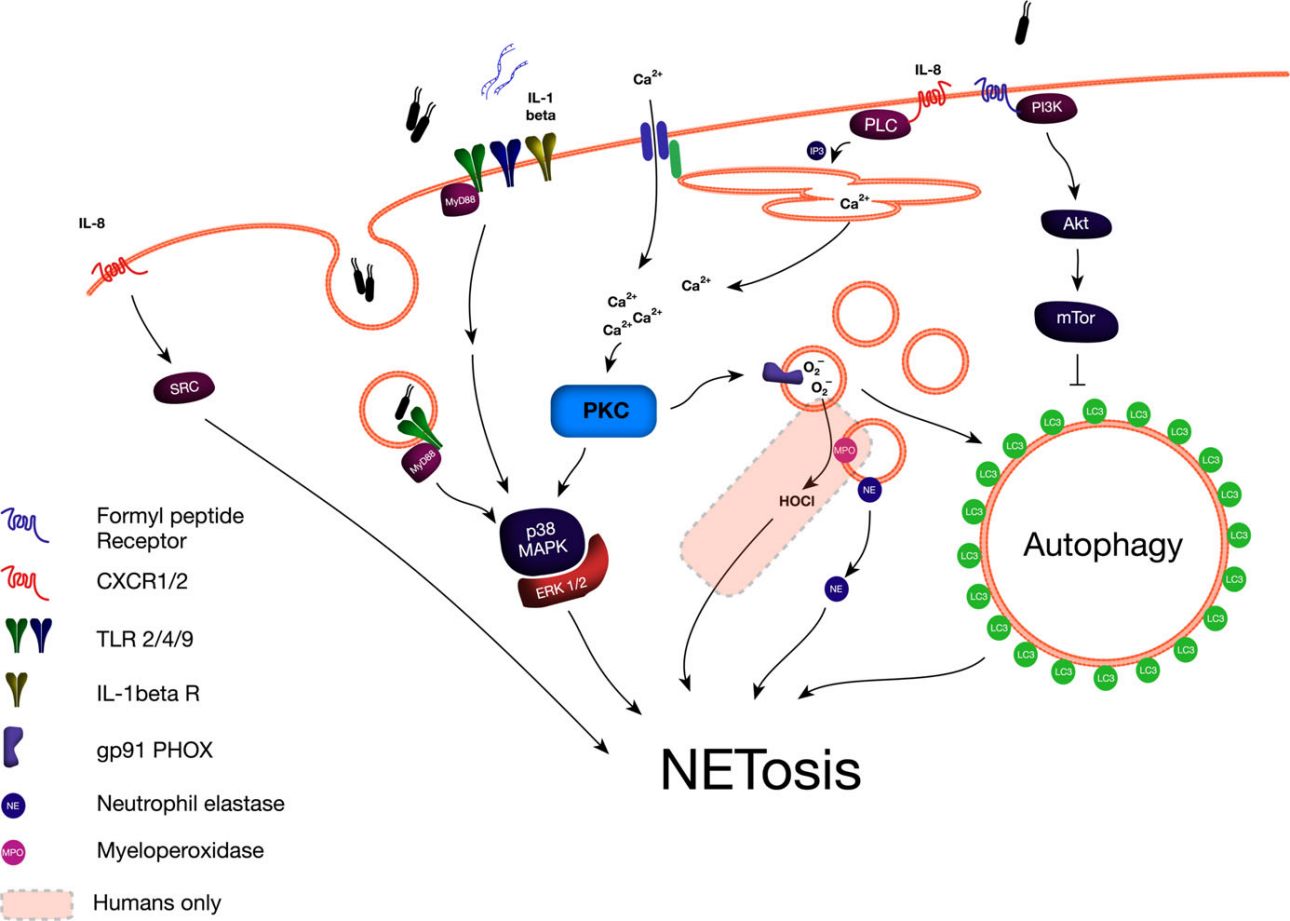
Signalling cascade leading to neutrophil extracellular trap release in murine and human neutrophils

Following signal detection, there are three critical steps leading to NET release: phagocyte oxidase/nicotinamide adenine dinucleotide phosphate‐oxidase (PHOX/NADPHO) activation, nuclear protease translocation and histone deimination (Figure 2).

Involvement of PHOX/NADPHO is illustrated by patients with chronic granulomatous disease, an inherited defect in PHOX activity, who are unable to produce NETs when stimulated with phorbol 12‐myristate 13‐acetate (PMA).^50^ This in turn leads to a clinical picture of recurrent and/or persistent infections, in particular with fungal pathogens. Impressively, there has been a successful report of gene therapy, where an 8‐year‐old boy was treated with a retroviral vector containing a functional gp91 (PHOX subunit) gene, resulting in neutrophils regaining the ability to NETose and leading to a termination of an intractable *Aspergillus nidans* infection.^51^ Experimentally, inhibition of NADPHO or myeloperoxidase (MPO) effectively inhibited NETosis stimulated by PMA, whereas inhibition of mitochondrial respiration or superoxide dismutase did not.^52^ PHOX/NADPHO is itself activated by protein kinase C (PKC). Pan‐activation of PKC isoforms using PMA or the di‐acyl glycerol analogue 1‐oleoyl‐2‐acetyl‐sn‐glycerol effectively stimulates NETosis.^53^ Specific inhibition of PKC isoform β inhibits both reactive oxygen species (ROS) production by PHOX and NETosis; however, there are conflicting reports on whether PKCζ is also able to inhibit NETosis.^54^ There are increasing reports of NADPHO‐independent NETosis, such as via the Rous sarcoma (src) kinase family in response to chemokine receptors (CXCR2) activation^55^ or via unspecified pathways following high‐dose uric acid stimulation.^56^ These reports highlight the deficiencies of investigating NETosis exclusively using PMA as the stimulant and demonstrate that while PKC activation is sufficient for the induction of NETosis, it is not the only pathway.

Histone deimination by peptidyl‐arginine transferase 4 (PAD4) is an essential step in NET release.^57^ PAD4 targets methyl‐arginine residues, reducing methylation and increasing citrullination on H4Arg3 and H3Arg2, 8 and 17 in HL‐60 cells^58^ over a time scale of 15 minutes to 2 hours, in a manner independent of caspase activity.^59^ These same post‐translational modifications are amongst the most immunogenic histone modifications seen in serum from patients with systemic lupus erythematosis,^60^ and levels of circulating nucleosomes and citrullinated histone H3 correlate with disease severity in acute inflammatory conditions including sepsis,^61^ trauma^62^ and pancreatitis.^63^ Genetic deletion of PAD4 leads to an inability of neutrophils to release NETs in response to calcium ionophore treatment or lipopolysaccharide (LPS),^64^ and pharmacological inhibition of PAD4 inhibits NET formation in murine and human neutrophils.^65^ Overexpression of PAD4, on the other hand, has been shown to cause histone hypercitrullination, nuclear decondensation and release of NET‐like structures in an osteosarcoma cell line.^66^

Nuclear translocation of granular proteases is the next step towards NET release. Neutrophil azoruphilic granules contain neutrophil elastase (NE), proteinase 3 (PR3) and cathepsin G (CG); however, only NE is translocated to the nucleus and neither inhibition of PR3 nor CG can prevent this translocation.^7^ Furthermore, the process does not appear to be mediated by fusion of granules with the nucleus, but rather NE dissociates from the granular membrane in a ROS‐dependent manner, before degrading cytosolic actin, arresting actin dynamics and translocating across the nuclear membrane using specific translocation mechanisms.^67^ Binding of nucleic acid by proteases initiates a process of degradation of nuclear binding proteins^68^ and controlled integration of MPO into the forming NET. Nuclear NE leads to early degradation of linker histone H1, followed by core histone H4 which coincides with nuclear chromatin decondensation.^7^ Histone H3 appears to be resistant to degradation in intact nuclei, but not in purified form, suggesting one of the purposes of post‐translational modification is to render histone H3 resistant to NE‐related degradation. This offers novel targets for therapy that have not yet been exploited.

The pathway described above is the best described due to the use of PMA as experimental stimulant of NETosis. In this experimental setup, the three steps are sequential; however, there have been recent reports of NET‐like structures being released rapidly (minutes), by budding of DNA/histone/protease‐containing vesicles from the nucleus followed by active exocytosis of NET‐containing vesicles.^69^, ^70^ This potentially bypasses most of the mechanisms described above and requires further study.

### 3.3 Autophagy

Although most studies support the conclusion that autophagy is essential for NETosis,^71^, ^72^ inhibition of mammalian target of Rapamycin (mTOR), a regulatory and inhibitory protein complex, has been reported to reduce NETosis stimulated by bacterial LPS.^73^ Stimulation of human neutrophils with vasculitis‐associated antibodies led to massive vacuolization, increased LC3BI degradation and could be reduced with the inhibitors of autophagy 3‐methyladenine (3MA) and LY294002.^74^ Similarly, LC3B containing vacuoles were observed preceding NETosis in LPS or septic plasma‐induced NETosis in human neutrophils which was also effectively inhibited by 3MA and bafilomycin A1.^75^ Knock down of phosphatase and tensin homologue deleted on chromosome 10 (PTEN), a potent regulator of autophagy, reduced PMA‐induced NETosis in HL‐60 cells and overexpression increased it.^76^ PKC has been shown to stimulate autophagy which in response to certain stimuli can be independent of mTOR, offering a potential explanation for this discrepancy.^77^ Figure 2 demonstrates how different stimuli resulting in NETosis can have differential effects on autophagy.

## 4 MOLECULAR BASIS OF HISTONE‐RELATED CELLULAR AND TISSUE INJURY

### 4.1 Effects of concentration and histone type on different cells and/or tissues

A wide variety of organisms actively release histones and histone degradation products as microbicidals (histone‐derived antimicrobial peptides; HDAP). Table 1 records a list of HDAPs, the species of origin and purported mechanism of antimicrobial action. The mechanisms of action appear to divide into membrane permeabilizing effects or DNA‐binding and disruption of transcription, which is why some HDAPs are also under investigation for the treatment of cancer. Full length core histones (H2A, H2B, H3 and H4) have shown antimicrobial activity in vitro^78^ in animal^79^ and human^80^ physiology and have the ability to neutralize bacterial endotoxin.

**Table 1:** Summary of histone‐derived anti‐microbial peptides (HDAPs), their species of origin and mechanism of action

Source histone	HDAP	Species of origin	Mechanism of action	References
H1	Full length	Coho salmon (*Oncorhynchus kisutch*)	Synergism with flounder pleuricidin. Mechanism unknown	81
H2A	Hipposin	Atlantic halibut (*Hippoglossus hippoglossus* L.)	Membrane permeabilization	82, 83
	Buforin I, II, III	Asian toad (*Bufo bufo gargarizans*)	DNA/RNA binding and disruption of cellular functions	84, 85
	Acipensins	Russian sturgeon (*Acipenser gueldenstaedtii*)	Outer membrane permeabilization	86
	Himanturin	Round whip ray (*Himantura pastinacoides*)	Unknown	87
	Abhesin	Disk abalone (*Haliotis discus discus*)	Unknown—possible inhibitor of transcription	88
	Parasin I	Catfish (*Parasilurus asotus*)	Membrane permeabilization	89, 90
H4	Full length	American cupped oysters (*Crassostrea virginica*)	Unknown	91
	Histogranin	Cow (*Bos taurus*)	DNA gyrase inhibitor	92
	MrH4	Freshwater giant prawn (*Macrobrachium rosenbergii*)	Unknown	93

Cellular injury mediated by extracellular histones has been described experimentally or in human disease of the lung,^94^ heart,^95^ liver,^96^ kidney^31^ and vascular endothelium.^62^ Table 2 details the effects of extracellular histones observed in specific cell types.

**Table 2:** Summary of effects of histones on different cell types of epithelial, endothelial and mesenchymal origin seen in in vitro and ex vivo experiments

Cell type	Effects of histones in vitro or ex vivo	Effective therapies
Epithelial		
A549,^97^, ^98^ BEAS‐2B,^99^ LA‐4,^94^ MLE‐12,^94^ mouse type II pneumocytes,^98^ L02 hepatocytes,^100^ CHO‐K1,^101^ CHO‐A745^101^	Calcium influx, cytokine (IL‐1β, IL‐6, IL‐10, TNFa) production (A549, BEAS‐2B, LA‐4) and cell death (PI/LDH; all cell types)	Anti‐histone antibodies, APC, heparin, polysialic acid, CIINH
Pancreatic acinar cells^102^, ^103^	Trypsin/Chymotrypsin activation, p‐STAT3/t‐STAT3 up‐regulation, cell death (PI)	Polysialic acid
HEK293,^96^, ^104^ parietal epithelial cells,^105^ podocytes^105^	Up‐regulated TLR2 and TLR4 expression, APC generation	
Endothelial		
HPMEC,^62^, ^98^ MLVEC^106^	Cell death (PI/LDH)	Anti‐histone antibodies, APC, heparin, CIINH
HCAEC^107^	Up‐regulation of tissue factor mRNA and extression and translation, NF‐kB/AP‐1 activation	
EA.hy926,^13^, ^14^, ^26^, ^62^, ^108^, ^109^ HUVEC^13^, ^14^, ^62^, ^107^, ^109^, ^110^, ^111^	Calcium influx, IkB depletion, p38MAPK/NF‐kB/AP‐1 activation, tissue factor and vWF generation/release, cell death (PI/AnnexinV binding)	Anti‐histone antibodies, APC, heparin, polysialic acid, CRP, MBP‐p33, PTX3
Glomerular endothelial cells^105^	TNFa mRNA expression, cell death (MTT)	Anti‐histone antibodies
Mesenchymal		
Murine cardiomyocytes,^112^ HL‐1 cardiomyocytes^113^	Cytosolic ROS production, calcium entry, mitochondrial impairment, reduced contractility, cell death (PI)	
Peripheral neutrophils,^46^, ^62^ HL‐60^101^	IL‐6 production, NETosis, cell death (PI)	Anti‐histone antibodies, IAIP, HMW‐HA
Peripheral monocytes,^23^, ^114^ MM6,^115^ U937,^100^ THP‐1^114^	Cytokine production (IL‐1β, IL‐6, IL‐8, IL‐10, TNFa, CXCL10), cell death (PI/LDH), factor Xa/tissue factor generation	Anti‐histone antibodies, heparin, CRP
Murine peritoneal macrophages,^116^ RAW264.7,^102^, ^107^ Kupffer cells,^33^, ^96^ J774 macrophages^105^	Inhibited clearance of other immune cells, HMGB1 secretion, TNFa production, increased tissue factor expression, vWF/angiopoietin‐2/P‐selectin release	APC
Human peripheral DCs,^34^ human monocyte‐derived DCs,^117^ murine BMDCs^105^, ^118^	TNFa production, NLRP3 protein up‐regulation, mitochondrial membrane dysfunction	Anti‐histone antibodies, APC, heparin
Human peripheral lymphocytes^119^	Apoptosis, p38‐MAPK phosphorylation, mitochondrial dysfunction, reduced bcl2 expression, caspase‐3 activation	
Platelets^14^, ^101^, ^108^, ^120^, ^121^, ^122^, ^123^	Calcium influx, platelet aggregation, thrombin generation, P‐selectin/factor Va expression	APC, heparin, CRP, HAS, IAIP, HMW‐HA
Human erythrocytes^47^, ^108^, ^124^	Haemolysis, procoagulant	APC, heparin, MBP‐p33

Breakdown of DNA in NETs with DNAse only partially ameliorates NET‐related toxicity, as it does not affect the histone component.^125^ Concentrations below 10 μg/mL seem to have a signalling function and can induce calcium transients in cells.^126^ Concentrations greater than 10 μg/mL (or 20 μg/mL in the presence of serum) induce cell death by an uncertain mechanism, which may involve the formation of non‐specific cationic pores in cell membranes.^127^, ^128^ Concentrations above 100 μg/mL cause rapid necrosis.

Core histones H3 and H4 are most frequently reported to increase in plasma from sepsis patients as well as experimental sepsis and therapeutic administration of antibodies to these histones improve outcomes in these models.^102^, ^129^, ^130^ It is conceivable that histone citrullination as described above renders these less susceptible to degradation and easier to detect, creating a publication bias. As core histones oligomerize readily with each other in solution^131^ and will surely rapidly do so upon histone release from any cell type, it is difficult to dissociate toxicity of individual histones from each other in biological systems. When used in in vitro studies, recombinant histones H2A and H2B were also able to induce cellular currents^126^ or activate thrombin.^122^

### 4.2 Calcium/ionic pore effects

The interaction of histones with cell membranes is heavily reliant on charge. Positively charged histones preferentially bind anionic phospholipids such as cardiolipin or phosphatidylserine, but not zwitterionic phospholipids like phosphatidylcholine.^132^ Furthermore, adding negative charge (eg, a phosphate head group as in phosphatidylinositol bis‐phosphate) increases the binding capacity of histones as measured by calorimetry.^128^ Histones have also been shown to expose phosphatidylserine on the surface of red blood cells in a dose‐dependent manner^47^; however, it is unclear whether this is as a result of altering flippase kinetics or via induction of apoptosis pathways. Once integrated, histones induce permeabilization of membranes to cations, disruptions of cellular calcium signalling^112^ and cell death by necrosis. Negatively charged acute‐phase proteins (such as C‐reactive protein, CRP),^14^ DNA,^128^ innate polysaccharides (heparin)^120^ or synthetic macromolecules^126^ compete with membrane phospholipids and prevent histone integration and toxicity. Bactericidal properties of histone fragments are dependent on their ability to form amphipathic α‐helices—potentially membrane spanning domains—however no such structural analyses have been performed on mammalian cells to date.^89^

### 4.3 Effects on coagulation

The ability of NETs and histones to influence the coagulation cascade and actually initiate venous thrombosis^8^, ^133^, ^134^ is the most recent detail in the emerging field of NETosis research. Clinically, circulating nucleosomes are independent prognostic markers of disseminated intravascular coagulopathy (DIC)^135^ and some countries, notably Japan, are actively promoting the use of anticoagulants as histone detoxification agents in DIC.^136^ Positive correlations between histone levels and coagulopathy can also be seen in trauma patients^137^ and patients with sepsis.^129^

**Figure 3:**
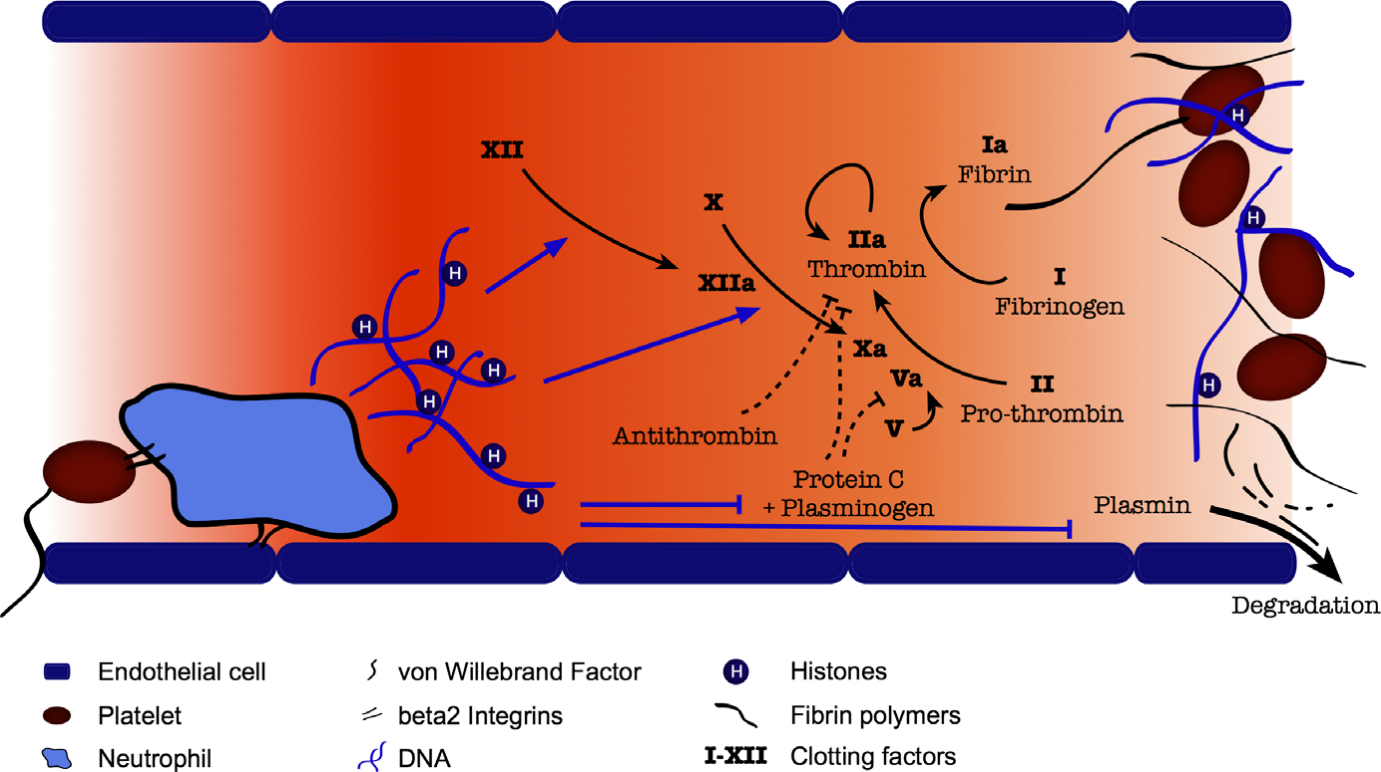
Interaction of histones and DNA with coagulation cascade to promote thrombosis

Figure 3 summarizes the effect of histones and NETs on the coagulation cascade. Histones act synergistically to produce a profound pro‐coagulant drive. Histones are able to induce platelet aggregation and factor V/Va expression and prothrombinase activity, leading to thrombin activation independent of the intrinsic coagulation pathway.^122^ Histones also inhibit thrombomodulin and protein C activation,^138^ an effect most pronounced with histones H3 and H4, thus reducing a natural thrombin inhibitor system. Furthermore, histone H4 binding promotes thrombin autoactivation, probably by fixing the prothrombin molecule in a conformational state conducive to proteolytic attack.^139^ The only exception is linker histone H1, which has been shown to reduce thrombin activation and prolong clotting times^140^; this mechanism is likely insignificant in acute inflammation, as histone H1 is amongst the first nuclear proteins to be degraded in the process of NETosis.

The presence of DNA in NETs also allows activation of the intrinsic coagulation pathway, demonstrated by NET‐enhanced thrombin generation in platelet‐poor plasma, reduced by factor XII/factor XI depletion or DNAse treatment.^141^ DNAse treatment in platelet‐rich plasma further increases thrombin generation, indicating differential effects of histones and NETs in different microenvironments. The addition of histones and DNA also increases fibrin fibre thickness, clot stability and delayed clot lysis^142^ as well as reducing anti‐thrombin‐mediated thrombin inactivation and plasmin activity.^143^ In in vivo systems, the interplay between von Willebrand factor (vWF), platelets and neutrophils anchors neutrophils to otherwise healthy vessel walls and permits NETing neutrophils to initiate clot formation,^125^, ^144^ with propagation that may occur or be enhanced by the mechanisms described above. Deficiencies in degradation of vWF produce clinical microangiopathies (eg, thrombotic thrombocytopenic purpura), the severity of which is also closely correlated with circulating NET components in humans.^145^

## 5 THERAPEUTIC STRATEGIES FOR HISTONE DETOXIFICATION IN PATHOLOGY

Histone toxicity is dependent on electrostatic membrane interaction with target cells. A number of innate and synthetic substances have demonstrated the ability to inhibit histone‐related toxicity based on surface charge alone, including plasma proteins (albumin,^123^ CRP^14^), polypeptides (polyglutamic acid^126^) and polysaccharides (heparin/heparanoids,^111^, ^146^ polysialic acid,^147^ bacterial O‐antigen^148^). Elevated histone‐degrading activated protein C (APC) levels are associated with better outcomes in sepsis^115^, ^149^ and trauma patients^137^; APC therapy is being evaluated for treatment of sepsis^150^ and pancreatitis.^151^ The effects of histones and NETs on the coagulation cascade can be overcome by therapy with thrombomodulin^121^ or tissue plasminogen activator (tPA),^143^ but the clot‐stabilizing effects of DNA in NETs must be overcome, which is well illustrated by the finding that DNAse therapy in addition to tPA is more effective than either therapy alone.^152^

In models of sterile and infective acute inflammatory diseases, administration of histones exacerbates end‐organ injury consistently (Table 3). Similarly, damaging effects are at least partly ameliorated by the application of histone‐targeted or histone‐specific therapies.

**Table 3:** Summary of effects of extracellular histones observed in in vivo models

Experimental model	Observations	Effective histone‐based treatment strategies	References
Sepsis			
Bacterial lipopolysaccharide (1‐40 mg/kg i.p/i.v.)	Elevation of circulating histones (including cit‐H3), leukocyte/platelet depletion/DIC; lung: neutrophil margination; endothelial vacuolization, intra‐alveolar haemorrhage and thrombosis; renal: cytokine/chemokine release, tubular apoptosis, neutrophil infiltration, death	APC, anti‐histone antibodies (H1, H4, pan‐histone), heparin (unfractionated or anti‐thrombin activity depleted), PTX3, PLD2 inhibition	13, 31, 95, 109, 111, 153, 154, 155, 156, 157, 158
Caecal ligation and puncture	Elevation of circulating histones, leukocyte apoptosis; lung injury; reduced cardiac output, left ventricular stroke volume and blood pressure (systolic and diastolic); cytokine release and injury of liver, kidney and spleen; death	Neutrophil depletion, Complement (C5aR1/C5aR2) receptor knock‐out, anti‐histone antibody, non‐anticoagulant heparin, PAD4 inhibition (Cl‐amidine)	13, 109, 112, 119, 154, 157, 159
MRSA (1‐10 × 10^7^ i.v.)	Bacterial dissemination in blood, liver, spleen, kidney and lung, with associated organ injury	Neutrophil depletion, unfractionated heparuin, DNAse I, vWF inhibition, PAD4 k/o, NE k/o or inhibition	125
Lung injury			
Bacterial lipopolysaccharide (1‐40 μg/animal i.t.)	Elevation of circulating histones; Pulmonary neutrophil infiltration, NETosis, elevated NE activity, abnormal gas exchange; death	Anti‐H4, aspirin, tirofiban, DNAse I, neutrophil depletion, C5a k/o	94, 97, 160
Intra‐nasal influenza A virus (10^2^ PFU) or *Streptococcus pneumoniae* (10^6^ PFU)	Elevation of circulating histones; pulmonary chemokine/cytokine release and inflammatory infiltrate	C1 esterase inhibitor	98
Intra‐tracheal irritant (HCl, 2 μL/g i.t. 0.01‐0.5 mol/L; Bleomycin 2.5 U/kg i.t.)	Elevation of circulating histones and DNA complexes; elevated pulmonary MPO/LDH activity, neutrophil infiltration, inter‐ and intra‐ alveolar oedema, reduced arterial oxygenation	Anti‐H4, heparin (unfractionated or N‐acetyl), C1 esterase inhibitor	98, 99, 161
Liver injury			
Ischaemia/reperfusion	Increase in hepatic H3 and H4 and cytokine release; increase in circulating histone‐DNA complexes	Anti‐H3/H4, PAD4 inhibitor	33, 46, 162
d‐galactosamine (300‐700 mg/kg i.p.) plus LPS (10‐40 mg/kg i.p.)	Hepatic leukocyte infiltration, hepatocellular apoptosis/necrosis; systemic cytokine release and transaminase elevation; death	Anti‐H4, antithrombin activity‐depleted heparin	100, 163
Acute pancreatitis			
Caerulein (50 μg/kg/h × 4 or 12 i.p.)	Elevation of circulating histones; pancreatic necrosis		163
Taurocholate (3.5%‐5% intra ductal)	Elevation of circulating and intra‐pancreatic histones and chemokines/cytokines; NETosis and inflammatory cell infiltrate within pancreas	Thrombin‐derived host defence peptides	63, 103, 164
l‐arginine (4 mg/kg i.p.)	Elevation of pancreatic histones, neutrophil infiltrate and oedema; pancreatic necrosis; death	Anti‐H3, thrombin‐derived host defence peptides	102, 164
Systemic administration of histones			
Calf‐thymus histones (0.75‐75 mg/kg i.v.)	Platelet depletion, haemolysis, elevation of vWF, fibrin and thrombin as well as systemic cytokines; prolonged bleeding time; pulmonary oedema, haemorrhage and microvascular occlusion; death	Heparin (unfractionated or O‐desulfated), C‐reactive protein, soluble thrombomodulin, anti‐histone antibody	14, 101, 104, 108, 110, 113, 120, 121, 165
Recombinant H3 (25‐100 mg/kg)	Leukocyte and platelet depletion; liver injury; death	Heparin (unfractionated and/or low molecular weight)	166

## 6 CONCLUSIONS

Histones and histone fragments are parts of an ancient antimicrobial mechanism conserved throughout eukaryotic species. In mammals, packaging of histones into NETs and interaction with the coagulation cascade presents an effective mechanism of limiting the spread of microorganisms and concentrating microbicidal peptides at a site of infection, but this comes at a cost of injury to adjacent tissue. In acute systemic inflammatory conditions, such as sepsis and trauma, systemic release of histones exacerbates micro‐circulatory thrombosis, worsens tissue perfusion and contributes significantly to organ injury. Recognition of this phenomenon may allow targeted therapy, limiting systemic injury and improving survival.

## CONFLICTS OF INTEREST

The authors confirm that there are no conflicts of interest.

## AUTHOR CONTRIBUTIONS

This review was designed by P.S. W.H and P.S undertook a systematic and comprehensive review of the literature, with critical input from A.V.T, D.N.C and R.S. All authors contributed to the critical review, editing and final approval of the manuscript.
